# Zinc Status Impacts the Epidermal Growth Factor Receptor and Downstream Protein Expression in A549 Cells

**DOI:** 10.3390/ijms23042270

**Published:** 2022-02-18

**Authors:** Emily Scheiermann, Mary-Ann Puppa, Lothar Rink, Inga Wessels

**Affiliations:** Institute of Immunology, Medical Faculty, RWTH Aachen University, Pauwelsstraße 30, D-52074 Aachen, Germany; emily.scheiermann@rwth-aachen.de (E.S.); mary-ann.puppa@rwth-aachen.de (M.-A.P.)

**Keywords:** zinc, EGFR, lung cancer, E-cadherin, PD-L1

## Abstract

Zinc has been suggested to play a role in carcinogenesis and tumor progression. Serum zinc levels of lung cancer patients are for example lower than in healthy individuals. The activation and expression of the epidermal growth factor receptor (EGFR), which plays a role in tumor biology, are presumably influenced by zinc. EGFR activation influences cell adhesion and immune escape. This study provides insights into the impacts of zinc on the EGFR activation and expression of downstream proteins such as E-cadherin and PD-L1 in the alveolar carcinoma cell line A549. To model chronic changes in zinc homeostasis, A549 cells were cultured in media with different zinc contents. EGFR surface expression of unstimulated and stimulated A549 cells was determined by flow cytometry. EGFR phosphorylation as well as the protein expression of E-cadherin and PD-L1 were analyzed by Western blot. In our hands, chronic zinc deficiency led to increased EGFR surface expression, decreased E-cadherin protein expression and increased PD-L1 protein expression. Zinc supplementation decreased EGFR surface expression and PD-L1 protein expression. In summary, zinc-deficient A549 cells may display a more malignant phenotype. Thus, future clinical research should further focus on the possible benefits of restoring disturbed zinc homeostasis, especially in lung cancer patients.

## 1. Introduction

Zinc is an essential trace element involved in various cellular processes such as DNA repair, receptor-induced signaling and enzyme stability. It, therefore, plays a particular role in proper immune function [1,2]. Zinc deficiency is a major public health problem and numerous diseases are promoted or influenced by a lack of zinc. Zinc deficiency can occur due to chronic inflammation and malnutrition, among others, and bears many health risks. These include a decreased sense of smell and taste, delayed wound healing, growth retardation and various chronic diseases [1,2,3,4].

Recently, a role in carcinogenesis was attributed to zinc deficiency [1,5,6,7,8,9,10]. Serum zinc levels of lung cancer patients are significantly lower in comparison to healthy controls [11,12,13,14]. In addition, the differential expression of certain zinc transporting proteins was associated with cancer [15,16,17]. However, the role of zinc in cancer development and progression is complex and needs to be further explored.

The epidermal growth factor receptor (EGFR) is an ErbB family receptor tyrosine kinase, which was found to contribute to tumorigenesis by supporting cell survival, proliferation, motility and other processes defining cancer cells [18]. The EGFR is also a main therapeutic target in lung cancer, since it is frequently overexpressed, exhibits amended signaling or is mutated in non-small cell lung cancer (NSCLC) [1,18,19,20]. Moreover, the EGFR promotes immune escape by tumor cells through the inhibition of the cytotoxic activity of T cells [21]. Zinc was suggested to activate the EGFR but the exact mechanism is currently unknown, although several hypotheses have been stated [22,23,24,25,26]. In addition, the effects of zinc on the EGFR may vary depending on the cell type and method of exposure. 

Recent studies suggested an impact of the EGFR and activation of its signaling cascades upon expression of proteins, which play a role in EMT (epithelial-to-mesenchymal transition) and immune escape, similarly to E-cadherin and programmed death ligand 1 (PD-L1), respectively [27,28,29,30]. E-cadherin is important for cell adhesion and downregulated in EMT. During EMT, the cellular phenotype changes from a polarized, epithelial phenotype towards a mesenchymal fibroblastoid phenotype, which is linked to cancer progression [31]. PD-L1, expressed by tumor cells, is an immune checkpoint molecule that can regulate T cell function, and thereby contributes to immune evasion by malignant cells [21,32]. This study provides insights into the impacts of alterations in the zinc status upon the EGFR activation and expression of EGFR-related proteins in A549 cells, a human lung cancer cell line that serves as a model for NSCLC.

## 2. Results

### 2.1. Alterations in Zinc Status Impact Epidermal Growth Factor Receptor Surface Expression

**Figure 1:**
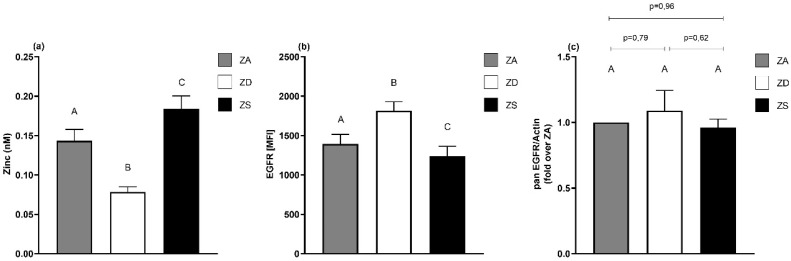
EGFR surface expression depends on cellular zinc content. A549 cells were cultured in zinc-adequate (ZA), zinc-deficient (ZD) or zinc-supplemented (ZS) medium for four days. Intracellular free zinc concentrations were analyzed using Zinpyr-1 via flow cytometry (**a**). Intracellular zinc contents are affected according to medium zinc content. Data for *n* = 6–10 independent experiments are shown as means + SEM. The EGFR surface expression of ZA, ZD and ZS cells was measured using flow cytometry (**b**). Zinc deficiency leads to an increased EGFR surface expression while zinc supplementation decreases the EGFR surface expression in A549 cells. Mean fluorescence intensities (MFI) of *n* = 11 independent experiments are presented as means + SEM. Total amounts of EGFR in ZA, ZD and ZS A549 cells were detected by Western blotting and normalized over β-actin (**c**). Data for *n* = 9 independent experiments are shown as means + SEM. Statistical significances between data sets were calculated using one-way ANOVA and Tukey’s post hoc test (*p* < 0.05). The same letter is never shared by statistically different data sets.

Initially, measurements of intracellular free zinc by flow cytometry confirmed that the cultivation of cells in zinc-deficient medium for four days resulted in a significant decrease, while cultivation in zinc-supplemented medium significantly increased the intracellular free zinc concentration (Figure 1a). Former research suggested that an acute increase in intracellular zinc levels leads to decreased EGFR surface expression [33]. To examine the influence of long-term changes in intracellular zinc homeostasis, the EGFR surface expression was measured by flow cytometry (Figure 1b). 

In line with the mentioned former findings, zinc supplementation resulted in a decreased receptor surface expression compared to the zinc-adequate control. Interestingly, zinc deprivation resulted in the opposite effect; the EGFR surface expression increased in comparison to the zinc-adequate control and zinc-supplemented cells. In conclusion, zinc supplementation decreased and zinc deprivation increased the EGFR surface expression (Figure 1b). In contrast to zinc’s effect on the EGFR surface expression, we did not detect significant changes in the total protein expression of the EGFR (Figure 1c).

### 2.2. Zinc Content in Media Affects mRNA Expression of Zinc Transporters and -Binding Proteins 

Cultivation in media with different zinc contents affects the intracellular free zinc concentration. Since zinc transport proteins, including ZIPs (Zrt- and Irt-like proteins) and ZnTs (zinc transporter), are well known to regulate the amount of intracellular zinc, we were eager to see if alterations in extracellular zinc homeostasis would affect zinc transporter expression. ZIPs lead to an intracellular zinc influx and normally show enhanced expression in zinc deficiency and decreased expression in zinc supplementation. ZnTs cause an efflux of zinc from the cytoplasm and are usually downregulated in cases of zinc deficiency and upregulated in zinc supplementation [34,35,36]. 

**Figure 2:**
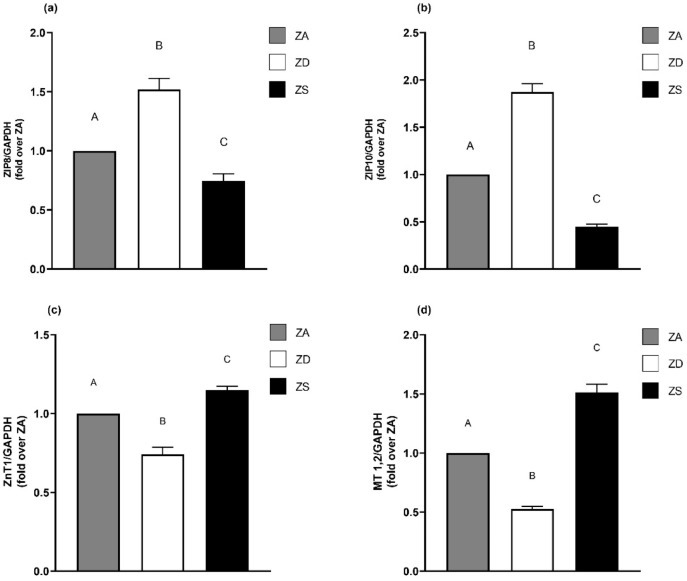
Zinc status impacts mRNA expression of zinc transporters and zinc-binding proteins. The mRNA expression levels of ZIP8 (**a**), ZIP10 (**b**), ZnT1 (**c**) and MT1,2 (**d**) in zinc-adequate (ZA), zinc-deficient (ZD) and zinc-supplemented (ZS) cells after four days of culture were detected by qRT-PCR. Expression after normalization over GAPDH of *n* = 6–8 independent experiments is shown as means + SEM. Statistical significances between data sets were calculated using one-way ANOVA with Tukey’s post hoc test (*p* < 0.05). Statistically different data sets do not share the same letter.

ZIP8, ZIP10 and ZnT1 were reported to be differentially expressed in lung cancer cell lines [37]. They were, therefore, chosen as representative transporters and their mRNA expression in A549 cells cultured in different media was measured. For both ZIP8 and ZIP10, zinc deficiency significantly increased mRNA expression, while zinc supplementation decreased mRNA expression (Figure 2a,b).

Zinc deficiency downregulated ZnT1 mRNA expression while zinc supplementation upregulated ZnT1 mRNA expression (Figure 2c). Metallothioneins (MTs) are the most important zinc-binding proteins and are involved in the regulation of intracellular zinc homeostasis [38]. MTs were reported to be downregulated in zinc deficiency and upregulated in zinc supplementation [35,39], which we verified for A549 cells in regard to MT1 and MT2 mRNA expression (Figure 2d).

### 2.3. Epidermal Growth Factor Receptor Phosphorylation Status Depends on Zinc’s Availability

**Figure 3:**
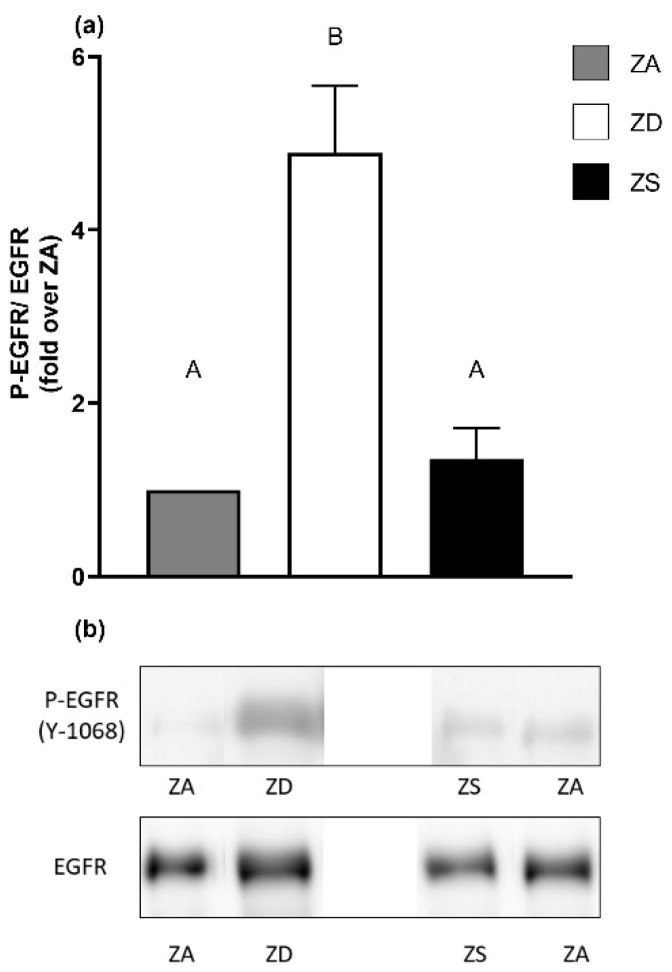
EGFR phosphorylation is increased in zinc deficiency. EGFR receptor phosphorylation at Y1068 in zinc-adequate (ZA), -deficient (ZD) and -supplemented (ZS) A549 cells was determined by Western blotting and normalized over the pan EGFR. Phosphorylation at Y1068 is five-fold increased in ZD cells compared to ZA cells. ZS cells exhibit comparable phosphorylation as ZA cells. Data for *n* = 9 independent experiments as means + SEM are depicted (**a**). One representative Western blot is shown (**b**). Statistical significances were calculated with one-way ANOVA and Tukey’s post hoc test (*p* < 0.05). Statistically different data sets do not share the same letter.

Next, we were curious to investigate whether the long-term changes in zinc homeostasis impacted the EGFR phosphorylation in a similar manner, as was observed for the receptor surface expression. Therefore, EGFR phosphorylation was analyzed by Western blotting. We measured phosphorylation at tyrosine 1068 (Y1068), a major autophosphorylation site of the EGFR [40] in the cells grown in medium with different zinc contents, as depicted in Figure 3. No difference in receptor phosphorylation was observed between zinc-adequate and zinc-supplemented cells. In contrast, the receptor phosphorylation of zinc-deficient cells was significantly increased in comparison to both zinc-adequate and -supplemented cells. In summary, only zinc deprivation led to activation of the EGFR illustrated by its phosphorylation (Figure 3).

### 2.4. Zinc Status Impacts mRNA and Protein Expression of Cancer-Relevant Targets

**Figure 4:**
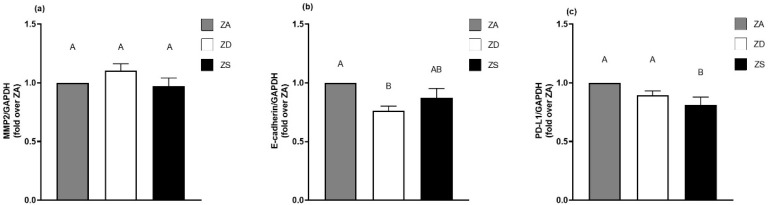
Zinc status impacts mRNA expression of E-cadherin and PD-L1. The mRNA expression levels of MMP-2 (**a**), E-cadherin (**b**) and PD-L1 (**c**) in zinc-adequate, zinc-deficient and zinc-supplemented cells after four days of cultivation in appropriate mediums were detected with qRT-PCR. Expression after normalization over GAPDH of *n* = 6 independent experiments are shown as means + SEM. Statistical significances between data sets were calculated using one-way ANOVA with Tukey’s post hoc test (*p* < 0.05). Statistically different data sets do not share the same letter.

Since we observed an impact of altered zinc homeostasis upon the surface expression and phosphorylation of the EGFR, we investigated whether the altered zinc homeostasis also affects the expression of the EGFR-regulated, cancer-relevant proteins E-cadherin, MMP-2 and PD-L1. E-cadherin is a cell adhesion protein expressed in adherens junctions. It plays a role in EMT during metastasis [28,31]. Matrix metalloproteinase 2 (MMP-2) [41] contributes to angiogenesis and tumor invasion [42]. The third cancer-relevant protein we examined was PD-L1, which participates in immune evasion [30]. The mRNA expression of E-cadherin, MMP-2 and PD-L1 was analyzed with qRT-PCR (Figure 4). MMP-2 expression was not affected by zinc. Therefore, the MMP-2 protein expression was not analyzed. Regarding E-cadherin, zinc deficiency led to a significant decrease in mRNA expression compared to the zinc-adequate control. Zinc supplementation also resulted in a slight decrease in E-cadherin mRNA expression, which was not significantly different from the zinc-adequate or zinc-deficient cells. Concerning the mRNA expression of PD-L1, both zinc supplementation and deprivation caused a decrease. However, only the decrease induced by zinc supplementation was significantly different from the zinc-deficient and -adequate cells.

**Figure 5:**
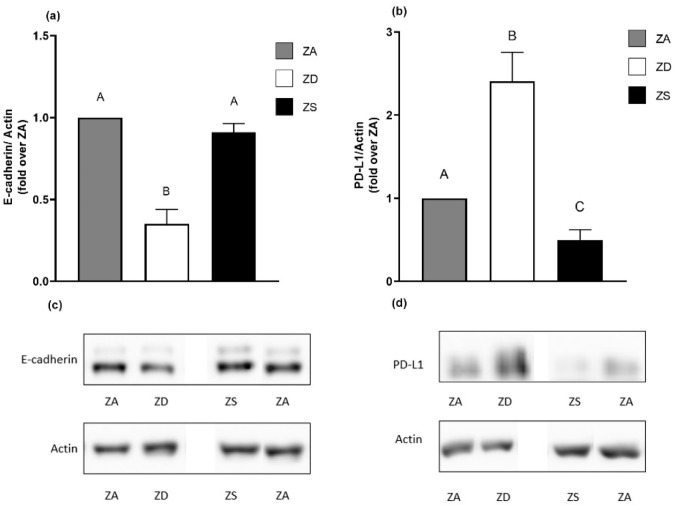
Zinc homeostasis influences protein expression of E-cadherin and PD-L1. The protein expression levels of E-cadherin (**a**) and PD-L1 (**b**) normalized over β-actin in zinc-adequate (ZA), zinc-deficient (ZD) and zinc-supplemented (ZS) cells were determined by Western blotting. Representative Western blots are presented (**c**,**d**). Means + SEM of *n* = 3–5 independent experiments are depicted. Statistically different data sets do not share the same letter (calculated by one-way ANOVA with Tukey’s post hoc test (*p* < 0.05)).

To confirm the PCR results, the protein expression levels of E-cadherin and PD-L1 in zinc-adequate, zinc-deficient and zinc-supplemented cells were compared using Western blotting (Figure 5). Reflecting the PCR results, zinc deficiency led to a significant decrease in E-cadherin protein expression compared to zinc-adequate and -supplemented cells, which was even stronger than the observed decrease in mRNA expression. Zinc supplementation did not impact the E-cadherin protein expression, which was in line with the slight decrease that we observed in the E-cadherin mRNA expression (Figure 5a).

Western blots revealed that PD-L1 expression (Figure 5b) was significantly decreased in zinc-supplemented cells compared to zinc-adequate and zinc-deficient cells, which our data for mRNA expression suggested. On the other hand, PD-L1 was significantly increased in zinc-deficient cells in comparison to zinc-adequate and -supplemented cells which differed from our data on mRNA expression. To summarize the above, zinc supplementation had no effect on E-cadherin but decreased the PD-L1 protein expression, while zinc deficiency led to decreased E-cadherin protein expression and increased PD-L1 protein expression in A549 cells (Figure 5).

### 2.5. EGFR Surface Expression Seems to Be Regulated by Membrane Fluidity Rather Than Receptor Phosphorylation

**Figure 6:**
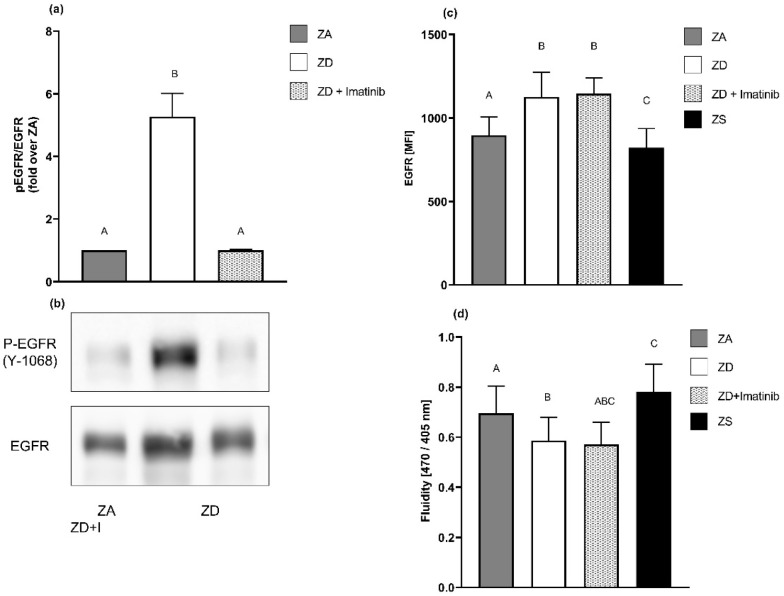
Zinc’s effects on membrane fluidity may explain changes in EGFR surface expression. A549 cell were grown in zinc-adequate medium (ZA), zinc-deficient medium (ZD), zinc-deficient medium with 0.1 µM imatinib (ZD+Imatinib) or zinc-supplemented medium (ZS) for four days. EGFR phosphorylation normalized over pan EGFR was measured by Western blotting (**a**). Representative Western blots are presented (**b**) for *n* = 3 independent experiments. Data are depicted as means + SEM. EGFR surface expression was determined by flow cytometry (**c**). Mean fluorescence intensity levels (MFI) of *n* = 4 independent experiments are presented as means + SEM. Membrane fluidity was analyzed by detecting changes in the spectral properties of 1-pyrendecanoidacid using a fluorescence multiplate reader (**d**). Data for *n* = 7 independent experiments are depicted as means + SEM. Statistical significances between data sets were calculated using one-way ANOVA and Tukey’s post hoc test (*p* < 0.05). The same letter is never shared by statistically different data sets.

To elucidate the relation between the EGFR receptor surface expression and its phosphorylation, we conducted experiments with the tyrosine kinase inhibitor imatinib. Imatinib is mostly known for its therapeutical application in diseases associated with aberrant tyrosine kinase activity, such as chronic myeloid leukemia [43]. We chose a non-toxic concentration of imatinib (0.1 µM), which was able to reverse the ZD-induced increased EGFR phosphorylation at tyrosine 1068 but did not interfere with the basal receptor phosphorylation displayed by zinc-adequate cells (Figure 6a,b). In contrast to normalizing EGFR phosphorylation, the addition of imatinib to zinc-deficient A549 cells did not reverse the increased EGFR surface expression that we describe in Section 2.1 (Figure 6c). Thus, EGFR phosphorylation may not be the major mechanism responsible for the increased EGFR surface expression in zinc-deficient A549 cells.

Aster et al. [44] found out that zinc impacts the membrane fluidity and receptor expression of the granulocyte macrophage-colony-stimulating factor receptor (GM-CSFR). Therefore, we analyzed membrane fluidity as an alternative underlying mechanism explaining the increased or decreased EGFR surface expression of zinc-deficient and -supplemented A549 cells. Zinc deficiency and zinc supplementation revealed significant but opposing effects on membrane fluidity. Treating zinc-deficient cells with imatinib did not reverse these effects (Figure 6d).

### 2.6. Zinc Status Influences the Metabolic Activity of A549 Cells and Their Interaction with Immune Cells 

**Figure 7:**
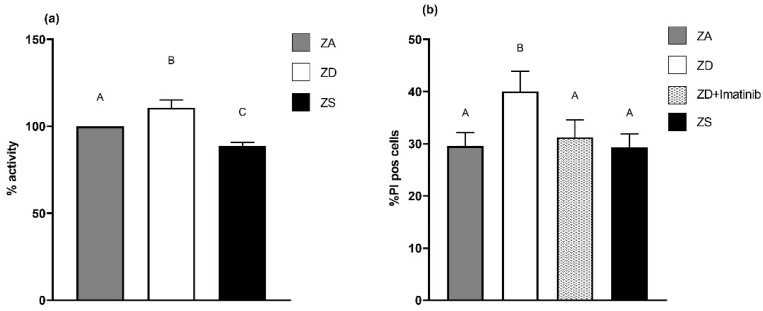
Zinc homeostasis affects the metabolic activity of A549 cells and their interaction with immune cells. Metabolic activity levels of zinc-adequate (ZA), zinc-deficient (ZD) and zinc-supplemented (ZS) cells were analyzed via MTT assay (**a**). Data for *n* = 10 independent experiments are shown as means + SEM. The amounts of PI-positive CD3^+^ cells from co-cultures with ZA-, ZD-, ZD+I (imatinib 0.1 µM)- and ZS-cultured A549 cells were determined by flow cytometry (**b**). Data for *n* = 17 independent experiments are presented as means + SEM. Statistical significances between data sets were calculated using one-way ANOVA and Tukey’s post hoc test (*p* < 0.05). The same letter is never shared by statistically different data sets.

Especially the increased expression of PD-L1 and decreased formation of E-cadherin indicated that zinc-deficient A549 cells may display a more malignant phenotype. Our data for zinc-treated A549 cells suggest that zinc supplementation may result in a less malignant phenotype. To support those hypotheses, we investigated whether the zinc status also impacts the metabolic activity of A549 cells, as a hint of their malignancy and the interaction between A549 cells and immune cells. Zinc deficiency significantly elevated metabolic activity in comparison to zinc-adequate cells, while zinc supplementation decreased general metabolic activity (Figure 7a). To gather a more functional insight into the effects of the increased PD-L1 protein expression of zinc-deficient A549 cells, we co-cultured A549 cells with PBMC (peripheral blood mononuclear cells) following the protocol of Chen et al. [30]. Since a direct relation between EGFR activation and PD-L1 expression has been reported [30], we also included zinc-deficient cells that were treated with the tyrosine kinase inhibitor imatinib. For PBMC, which were co-cultured with zinc-deficient A549 cells, we detected a significantly higher number of PI-positive CD3^+^ T cells. No significant differences were detected between CD3^+^ T cells from zinc-adequate and -supplemented cell co-cultures. Zinc-deficient cells that were treated with imatinib showed the same PI rates as zinc-adequate and -supplemented cells, but the numbers were significantly different from zinc-deficient cells (Figure 7b).

### 2.7. Establishment of a Model for Activated A549 Cells 

**Figure 8:**
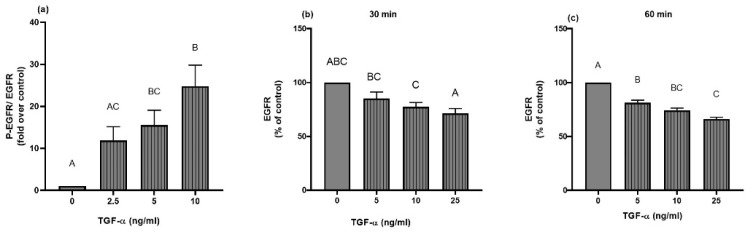
TGF-α induces EGFR phosphorylation and endocytosis. A549 cells were stimulated with different TGF-α concentrations (2.5 ng/mL, 5 ng/mL, 10 ng/mL) for one hour, then EGFR phosphorylation at Tyrosine 1068 was measured with Western blotting and normalized to pan EGFR (**a**). Data for *n* = 6 independent experiments are presented as means + SEM. EGFR surface expression was determined by flow cytometry upon stimulation with different concentrations of TGF-α (5 ng/mL, 10 ng/mL, 25 ng/mL) for 30 min (**b**) and 60 min (**c**). Data were normalized to the control sample. Data for *n* = 3 independent experiments are shown as means + SEM. Statistical significances between data sets were calculated by using one-way ANOVA and Tukey’s post hoc test (*p* < 0.05). The same letter is never shared by statistically different data sets.

We mentioned our suspicion that changes in zinc homeostasis may have an impact on the basal EGFR activity. To elucidate whether zinc homeostasis also exerted a further impact upon activated A549 cells, the cells were stimulated with epidermal growth factor (EGF) or transforming growth factor-alpha (TGF-α), both known to be natural ligands of the EGFR [45]. For NSCLC, the overproduction of EGFR ligands is also described [18,46], which presents another reason to investigate the effects of EGF and TGF-α. Barth et al. [33] established a phosphorylation and endocytosis model of the EGFR in A549 cells for stimulation with EGF, which we extended for stimulation with TGF-α. After one hour of stimulation, TGF-α led to dose-dependent EGFR phosphorylation at tyrosine 1068 (Figure 8a). A similar dose-dependent effect was observed for EGFR surface expression after 30 min and one hour of TGF-α stimulation (Figure 8b,c). For all concentrations of TGF-α, the EGFR surface expression was lower after one hour of stimulation than after 30 min. 

In our hands, EGF did not cause changes in the intracellular zinc level of A549 cells upon stimulation [33]. Similarly, no effects on intracellular zinc levels were observed after TGF-α stimulation with the applied concentrations (5, 10, 25 ng/mL) and incubation times (5, 15, 30 min). 

For further experiments, a TGF-α concentration of 5 ng/mL was applied. Both EGF and TGF-α were used to elucidate the effects of an altered zinc status on EGFR surface expression and phosphorylation. In essence, TGF-α causes EGFR phosphorylation and endocytosis (Figure 8) but does not induce changes in the intracellular zinc concentration, as observed for epidermal growth factor [33]. 

### 2.8. Zinc Status and Stimulant-Induced Alterations in EGFR Surface Levels

**Figure 9:**
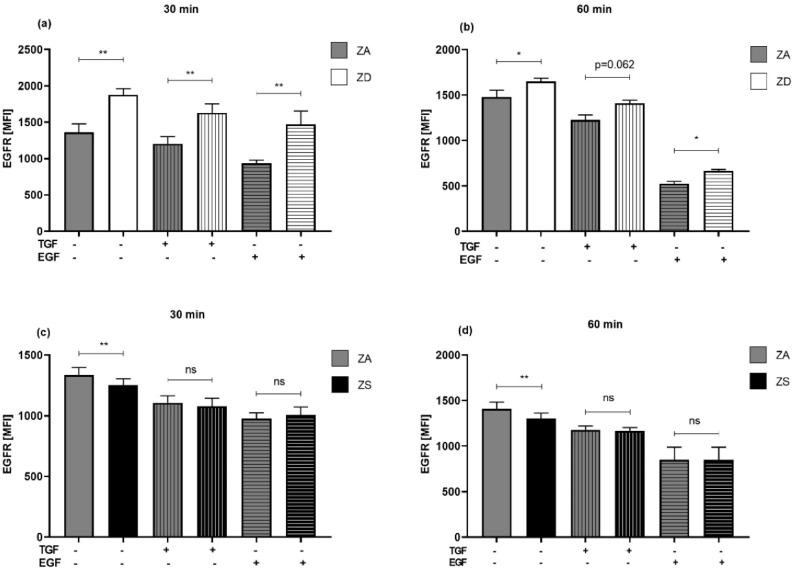
Effect of zinc status upon stimulation-induced EGFR endocytosis. A459 cells were pre-incubated with zinc-adequate (ZA), zinc-deficient (ZD) or zinc-supplemented (ZS) medium for four days. Subsequently, the effect of stimulation with TGF-α (5 ng/mL) or EGF (10 ng/mL) for 30 min (**a**,**c**) and 60 min (**b**,**d**) was detected by flow cytometry. Mean fluorescence intensity levels (MFI) were measured by flow cytometry. Data for *n* = 3–5 independent experiments are shown as means + SEM. Statistical significances, calculated by multiple student’s *t*-tests, are indicated as * (*p* < 0.05) and ** (*p* < 0.01); *p*-values ≥0.05 are depicted as not significant (ns).

Zinc-adequate, -deprived and -supplemented A549 cells were stimulated for 30 min or one hour with TGF-α or EGF. Subsequently, the EGFR surface expression was measured by flow cytometry. In general, receptor internalization was stronger after one hour of stimulation than after 30 min (Figure 9). Although unstimulated zinc-deficient cells revealed higher basal EGFR expression (Figure 1b), the extent of endocytosis did not differ between zinc-adequate and zinc-deficient cells (Figure 9a,b). As an example, after 30 min of stimulation with TGF-α, around 12% of the EGFR was internalized in both zinc-adequate and zinc-deficient cells (Figure 9a). For all time points and stimulants, the EGFR surface expression of zinc-deficient cells exceeded that of zinc-adequate cells. Regarding zinc supplementation (Figure 9c,d), we observed that after stimulation, the EGFR surface expression in zinc-supplemented cells matched the surface expression of zinc-adequate cells. However, since unstimulated zinc-supplemented cells revealed a lower basal expression level than unstimulated zinc-adequate cells, the extent of activation-induced endocytosis was lower in zinc-supplemented than in zinc-adequate cells. After 60 min of stimulation with EGF, for example, zinc-supplemented cells only internalized about 33% of the surface EGFR (Figure 9d), while zinc-adequate and -deficient cells internalized more than 50% of the receptor (Figure 9b). Thus, zinc-supplemented cells appear to respond more weakly to stimulants. In short, zinc deficiency does not alter the process of endocytosis, while zinc-supplemented cells exhibit a lower extent of activation-induced endocytosis (Figure 9).

### 2.9. The Zinc Status May Alter EGFR Sensitivity towards Stimulants

**Figure 10:**
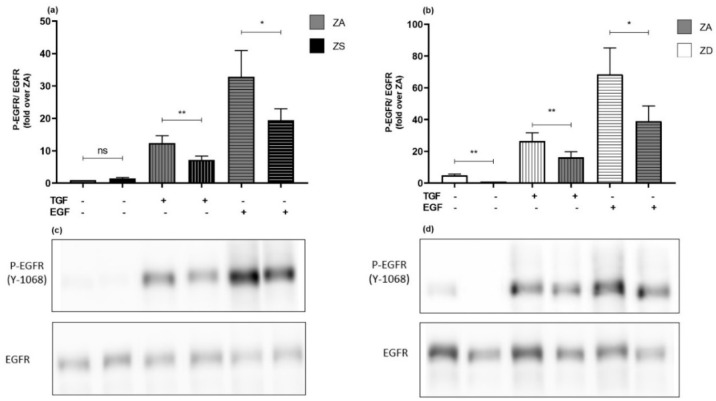
Zinc homeostasis alters cellular sensitivity towards stimulants. A549 cells pre-incubated in zinc-adequate (ZA), zinc-deficient (ZD), or zinc-supplemented (ZS) medium for four days were stimulated with TGF-α (5 ng/mL) or EGF (10 ng/mL) for 1 h. EGFR phosphorylation at tyrosine 1068 normalized to pan EGFR was determined using Western blotting (**a**,**b**). One representative blot is presented in (**c**) for ZA and ZS cells and in (**d**) for ZA and ZD cells. Data of *n* = 11 independent experiments are shown as means + SEM. Statistical significances, calculated by multiple student’s *t*-test, are indicated as * (*p* < 0.05) and ** (*p* < 0.01); *p*-values ≥ 0.05 are depicted as not significant (ns).

Since our data indicate an effect of zinc homeostasis on basal levels of receptor expression and phosphorylation, we analyzed the effects of the zinc status on activation-induced receptor phosphorylation. Therefore, zinc-adequate, zinc-deficient and zinc-supplemented A549 cells were stimulated with EGF or TGF-α for one hour and were then processed for detection of phosphorylation by Western blotting, as depicted in Figure 10. For all experimental settings, both stimulants induced phosphorylation at tyrosine 1068. For zinc-supplemented cells, phosphorylation levels caused by TGF-α and EGF were significantly lower than in zinc-adequate cells. In contrast, phosphorylation in zinc-deficient cells was significantly stronger than in zinc-adequate cells for both stimulants. Ultimately, our data suggest that zinc-supplemented cells respond more weakly towards stimulants, while activation of the EGFR in zinc-deprived cells may be augmented (Figure 10). 

### 2.10. Effects of Stimulation with Growth Factors on the Expression of Downstream Targets

**Figure 11:**
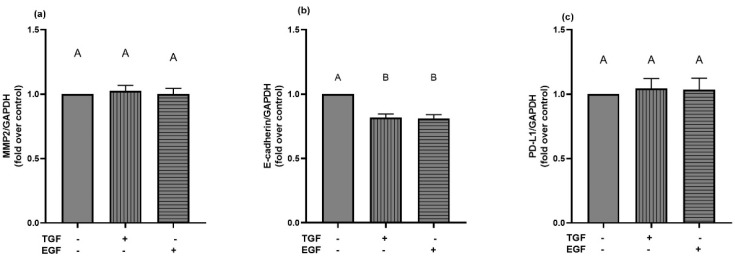
Stimulation with TGF-α and EGF leads to decreased E-cadherin mRNA expression. Zinc-adequate A549 cells were stimulated with TGF-α (5 ng/mL) or EGF (10 ng/mL) for six hours, and mRNA expression levels of MMP-2 (**a**), E-cadherin (**b**) and PD-L1 (**c**) were determined by qRT-PCR. Expression after normalization over GAPDH of *n* = 6–9 independent experiments is depicted as means + SEM. Statistical differences between data sets were calculated using one-way ANOVA with Tukey’s post hoc test (*p* < 0.05). Statistically different data sets never share the same letter.

Former research [28,30,41], as well as our own data, suggested a link between the phosphorylation of the EGFR; the subsequent activation of signaling pathways; and the expression of E-cadherin, MMP-2 and PD-L1. Therefore, we examined the mRNA expression of these genes after stimulation with TGF-α and EGF (Figure 11). The six hour stimulation did not lead to any significant changes in the mRNA expression of MMP-2 and PD-L1. 

For E-cadherin, both TGF-α and EGF caused significant reductions in mRNA expression in zinc-adequate cells. Interestingly, pre-incubation of A549 cells in the different zinc conditions did not lead to any changes in TGF-α and EGF-induced E-cadherin mRNA expression. 

**Figure 12:**
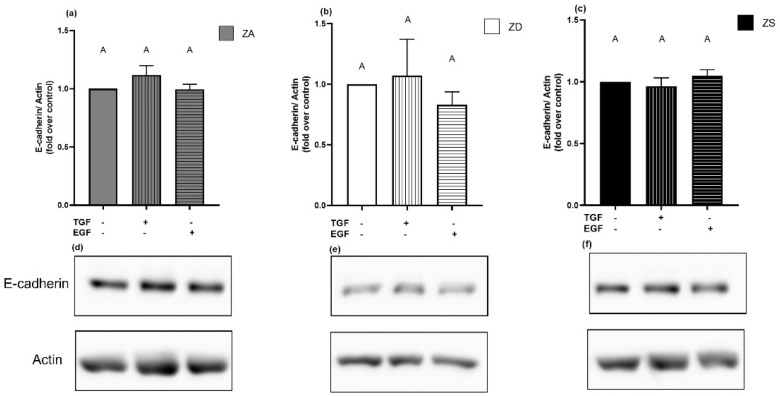
E-cadherin protein expression remains unaffected by stimulation. Zinc-adequate (ZA) (**a**), zinc-deficient (ZD) (**b**) and zinc-supplemented (ZS) (**c**) cells were stimulated with TGF-α (10 ng/mL) or EGF (20 ng/mL) for six hours and total E-cadherin protein expression normalized to β-actin was detected by Western blot. Data for *n* = 6 independent experiments are shown as means + SEM and respective representative blots are presented (**d**–**f**). Statistical differences between data sets were calculated using one-way ANOVA with Tukey’s post hoc test (*p* < 0.05). Statistically different data sets never share the same letter.

To validate the PCR results, we checked the protein levels of E-cadherin in stimulated cells previously grown in different zinc conditions. We could not confirm the effects of the stimulation-induced decrease in E-cadherin expression in zinc-adequate cells on protein level. In fact, the stimulation with TGF-α and EGF did not cause changes in the protein expression of E-cadherin in any of the zinc conditions at the times tested (Figure 12). In summary, none of the stimulants caused any noteworthy changes in mRNA or protein expression of the targeted cancer-relevant factors.

### 2.11. Zinc Deficiency Phosphorylates the EGFR, Elevates Its Surface Expression and Increases Metabolic Activity in PC-9 Cells

**Figure 13:**
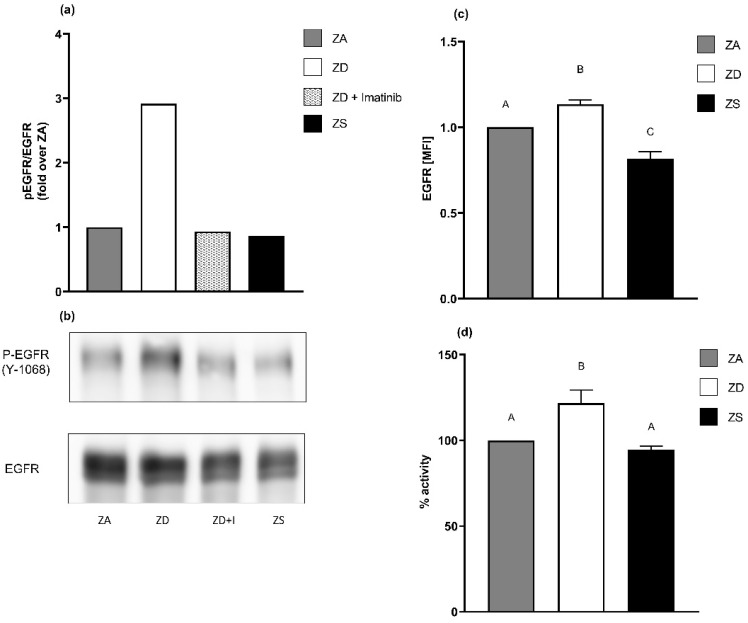
PC-9 cells were cultivated in zinc-adequate (ZA) medium, zinc-deficient (ZD) medium, ZD medium treated with 0.1 µM imatinib (ZD+Imatinib) or zinc-supplemented (ZS) medium for four days. EGFR phosphorylation normalized to the pan EGFR was detected in ZA, ZD, ZD+I and ZS PC-9 cells by Western blotting (**a**). A representative Western blot is shown in (**b**). Data for *n* = 1 experiment is shown as an outlook. EGFR surface expression levels of ZA, ZD and ZS PC-9 cells were measured by flow cytometry (**c**). Mean fluorescence intensity levels (MFI) of *n* = 4 independent experiments are shown as means + SEM. Metabolic activity levels of ZA, ZD and ZS PC-9 cells were analyzed via MTT assay (**d**). Data for *n* = 3 independent experiments are shown as means + SEM. Statistical differences between data sets were calculated using one-way ANOVA with Tukey’s post hoc test (*p* < 0.05). Statistically different data sets never share the same letter.

A549 cells serve as a model for NSCLC and express the wild-type EGFR. In lung cancer patients, the EGFR is often mutated [18]. Therefore, we decided to verify our key findings regarding the effects of an altered zinc status in PC-9 cells. The EGFR is mutated in PC-9 cells, making them supposedly more malignant [27]. Regarding the EGFR phosphorylation at tyrosine 1068, zinc-deficient PC-9 cells showed higher EGFR phosphorylation than zinc-adequate, -supplemented and imatinib-treated zinc-deficient PC-9 cells (Figure 13a,b). The EGFR surface expression was increased in zinc-deficient PC-9 cells in comparison to zinc-adequate PC-9 cells, while zinc-supplemented PC-9 cells expressed less EGFR on their surfaces than zinc-adequate and -deficient PC-9 cells (Figure 13c), as observed for A549 cells. The general metabolic activity of zinc-deficient PC-9 cells was significantly increased in comparison to zinc-adequate and -supplemented cells, as we found for A549 cells as well (Figure 13d).

## 3. Discussion

A previous study addressed the possible impact of an acute increase in the intracellular zinc concentration upon the activation, phosphorylation and endocytosis of the epidermal growth factor receptor (EGFR) [33]. To extend our previous findings, we modeled chronic changes in zinc homeostasis and included zinc supplementation as well as zinc deficiency in the study presented here. Zinc deficiency is frequently observed in around 30% of the world’s population and poses a global health problem [47]. By cultivating A549 cells [48,49] in culture media with different zinc contents, we achieved a more physiological and long-term alteration of the intracellular labile zinc concentration than in our previous study [33]. The literature suggests that the serum zinc levels of patients diagnosed with NSCLC are lower than in a healthy collective [11,12,13,14]. However, neither an association of altered serum zinc and disease etiology nor the possible benefits of normalizing serum hypozincemia are clearly defined yet. As a further step towards increasing our understanding of the role of zinc in NSCLC, we now provide insights into the impact of long-term changes in zinc status upon the EGFR and the expression of cancer-relevant proteins in pulmonary carcinoma cells. 

Regarding our results for the mRNA expression of zinc transporters and -binding proteins, we can confirm the previous findings [35,36] that zinc deficiency induces the expression of certain ZIPs, while the expression levels of MT1, MT2 and certain ZnTs are decreased in zinc-deficient cells. The opposite effects were observed after zinc supplementation. Thus, our results offer further proof that extracellular changes in zinc homeostasis are translated into an intracellular response. Together with the alterations in the intracellular zinc content, which was achieved depending on the extracellular zinc conditions, the effects described subsequently are probably related to chronic zinc deficiency or high zinc levels in the A549 cells.

Barth et al. [33] described a decrease in EGFR surface expression caused by an acute increase in intracellular zinc concentration. Our results extend this finding for long-term changes; the increase in intracellular zinc concentration led to a stable decline in receptor surface expression. Zinc deficiency led to the exact opposite and increased the receptor surface expression (Figure 1), which has not been described before.

Short-term and strong increases in intracellular zinc concentrations have been shown to elicit phosphorylation of the EGFR [22,24,33], yet the exact underlying mechanism remains unknown, although many hypotheses have been stated; for example, the direct influence of zinc on the receptor tyrosine kinase may be involved. Alternatively, zinc has been reported to inhibit certain phosphatases, which may explain the results reported by others [23]. 

Surprisingly, we did not observe increased receptor phosphorylation after long-term zinc supplementation, but rather after long-term zinc deficiency. Thus, zinc’s function as a phosphatase-inhibiting agent does not explain our data. It may be possible that the magnitude of the increase in intracellular zinc in this study is too low, and that the cells adapt quickly to the higher zinc concentration and maintain basal receptor phosphorylation. In a recent study [50], the protein tyrosine phosphatase receptor types G and J (PTPRG/J) were identified as mainly responsible for the dephosphorylation of the EGFR at the plasma membrane. So far, it is unknown whether zinc impacts these phosphatases. However, we cannot completely exclude a possible effect of zinc supplementation or deficiency on PTPRG/J. However, since treatment of zinc-deficient A549 cells with the tyrosine kinase inhibitor imatinib reversed the increased level of EGFR phosphorylation, which we measured in zinc-deficient cells, an elevated activity of tyrosine kinases may be a more likely mechanism responsible for this effect. Further research is needed to exactly understand the way in which zinc acts upon the EGFR phosphorylation and whether the effects of zinc on tyrosine phosphatases or kinases play a role in this regard. 

The EGFR represents an important receptor in cancer biology. It is often mutated, shows amended signaling, exhibits enhanced expression in cancer and is a therapeutical target in NSCLC [18,20,46]. Thus, we decided to examine cancer-relevant targets that are influenced by the EGFR according to the literature to explore a possible role of zinc in carcinogenesis [1,5,6,7,8,9,10]. Zou et al. proposed that EGFR activation leads to upregulation of MMP-2, which then caused degradation of E-cadherin in a squamous carcinoma cell line [28]. Yan et al. reported a decrease in E-cadherin caused by EGFR activation independently of MMP-2 [29]. Therefore, we examined both MMP-2 and E-cadherin expression. For MMP-2, we could not detect any changes in mRNA expression induced by zinc status. Regarding E-cadherin, we show that zinc deficiency leads to decreased mRNA and protein expression. These results lead us to the conclusion that zinc homeostasis affects the E-cadherin expression independently of MMP-2 in A549 cells. The relationship between zinc and cadherins has previously been discussed in the context of barrier function and cytoskeletal stability. Finamore et al. reported a disrupted barrier function induced by the impacts of zinc deficiency upon adherens junctions [51]. Thus, an influence of zinc on E-cadherin was suspected in intestinal Caco-2 cells, but no protein downregulation has been reported so far. Another study focused on the relationship between the EGFR, E-cadherin and the actin cytoskeleton. It was revealed that stimulation with EGF induced the dissociation of E-cadherin complexes from actin, stressing the role of EGFR signaling in cell–cell adhesion and tumor progression [52]. Interestingly, other studies have reported a reciprocal relationship between the EGFR and E-cadherin [53,54]. Therefore, further research should address this proposed feedback regulation. Our results are also in line with a study by Bao et al. [55], who reported increased shedding of E-cadherin by caspases during zinc deficiency in primary lung cells. As the decrease in E-cadherin is a prominent feature among EMT, our results suggest that zinc deficiency may promote events that result in EMT. A study by Ninsontia et al. stated that zinc induces EMT [56], which seems to oppose the data shown here. However, our study only provides insights into one feature of EMT, the E-cadherin expression, and we did not analyze the EMT process in total. Additionally, different time points of zinc stimulation and different cell lines were assessed by Ninsontia et al., which may explain the contradicting results. A functional study on EMT and zinc stimulation after four days of culture may bring clarification. Overall, our results suggest that zinc deficiency leads to activation of the EGFR and EGFR-induced genes, which is in accordance with previously published findings. Our data extend the understanding of zinc’s impacts upon the EGFR and downstream cancer-relevant proteins. 

According to the literature, the EGFR participates in immune evasion in human cancer, for example by expediting aerobic glycolysis [21]. Therefore, we focused on targets driving immune evasion. Programmed death-ligand 1 (PD-L1) is an immune checkpoint molecule expressed by immune-regulating cells [57], but also by malignant cells, which can thereby regulate T cell function and contribute to immune evasion [32]. Antibodies blocking the binding of PD-L1 to its receptor PD-1R are promising drugs for cancer therapy [30]. Moreover, PD-L1 has been reported to be an EGFR-regulated gene [27,30]. Furthermore, high PD-L1 expression in human lung cancer is associated with poor prognosis [58]. Recently, a link between EMT and the expression of PD-L1 has been reported in NSCLC [59]. Regarding zinc and PD-L1 expression, it was found that zinc promotes PD-L1 expression in dendritic cells during fungal infections [57]. Here, we report that chronic changes in extracellular zinc levels highly impact the PD-L1 protein expression in A549 cells in an opposing manner; zinc deficiency promotes PD-L1 expression, while zinc supplementation decreases the PD-L1 expression. Thus, zinc may have opposing effects on tumor cells compared to healthy immune cells. 

The results presented here are in line with the proposed increase in EMT in tumor patients, where PD-L1 is upregulated and E-cadherin is downregulated [27,31]. We observed both an increase in PD-L1 and decrease in E-cadherin in zinc-deficient cells. Thus, we propose the hypothesis that zinc deficiency may promote EMT while zinc supplementation seems to inhibit EMT, which requires further validation. 

Zinc’s importance for proper immune functions, especially during cancer, is well accepted [2,3,60]. Our data suggest that zinc is not only vitally important for immune cells but that a lack of zinc may also favor immune evasion by cancer cells. On the other hand, restoring a zinc sufficient status may not only re-establish an effective immune response but may also ameliorate malignancy of tumor cells. Clinical studies would be needed to illuminate the role of zinc and zinc deficiency—particularly in the metastatic progression of NSCLC but also in disease origin—and to establish zinc status as a marker for prognosis. Further research is needed to elucidate the association between upregulated PD-L1 in zinc-deficient A549 cells and the attenuated immune response that we found here.

Although the EGFR surface expression varies depending on the zinc status, Western blots revealed constant levels of total cellular EGFR. Thus, receptor degradation can rather be excluded as an explanation for the zinc-induced changes in EGFR surface expression, and translocation to the endosomes is likely [46]. To elucidate the underlying mechanism behind zinc’s impact upon EGFR surface expression, we measured the membrane fluidity. Moreover, we treated zinc-deficient cells with the tyrosine kinase inhibitor imatinib to explore the impact of zinc deficiency on receptor phosphorylation in relation to receptor expression. Treating zinc-deficient cells with imatinib reversed the elevated EGFR phosphorylation level, whereas the increased EGFR surface expression was not reversed by imatinib. Furthermore, we found that zinc homeostasis exerts opposing effects upon A549 membrane fluidity, which could not be reversed by imatinib. From these findings, we conclude that receptor phosphorylation and its surface expression are regulated independently, at least in regard to zinc homeostasis. Previous results from our group support this conclusion [33]. 

Since the zinc-induced changes in membrane fluidity were correlated to zinc-induced alterations in EGFR surface expression, we hypothesize that zinc’s effects on the membrane fluidity may be responsible for the increased or decreased EGFR surface expression. A similar association between zinc, membrane fluidity and receptor surface expression has been described before [44]. 

We hypothesize that changes in zinc status may affect the malignant cellular phenotype of A549 cells. To more deeply explore this hypothesis, we conducted two preliminary functional studies to shed light on this possible conclusion. As investigated by MTT test, zinc deficiency increases while zinc supplementation decreases metabolic activity, which supports our suggestion. To directly elucidate the effects of the more malignant phenotype that we propose for zinc-deficient cells, we co-cultured zinc pre-conditioned A549 cells with PBMC. Subsequently, the amount of PI^+^ CD3^+^ T cells was assessed. Co-culturing PBMC with zinc-deficient A549 cells led to elevated death of T cells. A similar effect was described by Chen et al. [30]. It is well known that increased PD-L1 expression induces cell death, especially in T cells, via interaction with their PD-1R, which supports immune escape by malignant cells [32]. Our results for the co-cultures reflect this mechanism. Moreover, treating zinc-deficient cells with imatinib, the tyrosine kinase inhibitor, succeeded in reversing the effect of zinc-deficiency-induced EGFR phosphorylation and also reversed the increased PBMC death rate. This suggests an association between zinc-dependent EGFR phosphorylation, PD-L1 protein expression and interaction with CD3^+^ cells. However, zinc has manifold effects on intracellular signaling, gene transcription, translation and protein stability. Thus, in addition to zinc’s effect on EGFR phosphorylation, other mechanisms may underly the changes in PD-L1 expression that we observed in zinc-deficient A549 cells. 

To examine other properties of NSCLC, we investigated the effects of two natural EGFR ligands. Since ligand overproduction is reported among NSCLC and overexpression of TGF-α is even associated with worse prognosis [18], we determined whether the effects we obtained for changes in zinc homeostasis augmented the effects of EGF and TGF-α [45]. In our hands, neither EGF nor TGF-α caused zinc flux in A549 cells. The phosphorylation of the EGFR induced by EGF or TGF-α stimulation may, thus, be independent from the intracellular zinc concentration. 

Since zinc-deficient cells expressed significantly more EGFR on their surface than zinc-adequate A549 cells, a higher absolute number of EGFR molecules is prone to ligand binding of TGF-α and EGF, as well as internalization. Moreover, the relative amounts of internalized EGFR were equal between zinc-deficient and -adequate cells. Thus, zinc deficiency may not have an impact on stimulation-induced endocytosis. We found that although zinc-supplemented cells express less EGFR on their surface, stimulation results in a final EGFR surface expression level that is comparable to the levels found in activated zinc-adequate cells. This finding leads us to the conclusion that zinc supplementation alters the cells’ responsiveness towards stimuli. However, it appears that a certain level of EGFR surface expression is not undershot, even if the basal EGFR surface expression is lower. 

Regarding the analysis of EGFR phosphorylation at tyrosine 1068, stimulated zinc-deficient A549 cells showed a stronger response, while zinc-supplemented cells revealed a weaker response. This effect could partly be explained by the fact that more or less of the receptor is initially expressed on the cell surface and prone to ligand binding. However, since EGFR surface expression in activated zinc-supplemented cells matches the surface expression of zinc-adequate cells after one hour of stimulation, this consideration cannot fully explain the observed effect. Regarding zinc deficiency, a greater amount of the receptor can be activated by ligands. This may explain the stronger activation-induced phosphorylation of the EGFR in zinc-deficient A549 cells. 

Former studies hinted towards an impact of EGFR activation on the MMP-2 activity in pancreatic cells after 24 h of stimulation with EGF [61]. However, we did not observe increased expression of MMP-2 in A549 cells after six hours, nor after 24 h of stimulation. Regarding PD-L1, Zhang et al. reported increased mRNA and protein expression upon EGF stimulation in HCC827 and PC-9 cells (both lung cancer cell lines). They also stated that the extent of PD-L1 expression correlates with EGFR mutations. The group, therefore, focused on cell lines with EGFR mutations, whereas A549 cells, which express a wild-type EGFR, were not further investigated [27]. In another study, Chen et al. found only relatively small amounts of PD-L1 in A549 cells and did not take this cell line into further consideration either [30]. Regarding E-cadherin, stimulation with EGF or TGF-α caused decreased mRNA expression, but no effect on the protein level was perceived in our study. A similar effect has been described before [62].

As a perspective for future studies, we investigated the effects of chronic zinc changes on PC-9 cells, another model for NSCLC, with a mutated EGFR [27]. EGFR mutations are generally associated with a more malignant phenotype [63]. Our first data for PC-9 cells revealed similar effects of chronic changes in zinc homeostasis regarding EGFR surface expression, EGFR phosphorylation and metabolic activity for A549 cells. These results suggest that the effects of an altered zinc status may affect EGFR surface expression and phosphorylation, as well as cellular metabolism, independent of existing EGFR mutations. Moreover, the effects of chronic changes in zinc homeostasis may not be limited to A549 cells.

In essence, zinc homeostasis impacts EGFR expression, phosphorylation and expression of downstream EGFR targets. In contrast, the zinc status appeared to not affect the activation-induced expression of downstream proteins. Moreover, we conclude from our data that the effects of alterations in zinc homeostasis upon E-cadherin and PD-L1 expression in non-stimulated cells cannot be entirely explained by zinc’s impact on the EGFR. In fact, changes in PD-L1 expression and metabolism may additionally be attributed to the many other cellular processes that are regulated by zinc [64]. We propose that the zinc status impacts on the epidermal growth factor receptor’s activity and surface expression but that the zinc status also acts independently of the EGFR upon its targets. EGFR receptor activation and zinc homeostasis may have synergistic instead of only agonistic effects upon E-cadherin and PD-L1 protein expression. Our findings indicate that the EGFR surface expression, phosphorylation and sensitivity towards stimulants are some of the cellular processes influenced by the zinc status but are not the only ones that impact E-cadherin and PD-L1.

In conclusion, zinc deficiency may lead to a more malignant cellular phenotype in NSCLC. Clinical research should further focus on the possible benefits of restoring disturbed zinc homeostasis in patients with NSCLC.

## 4. Materials and Methods

### 4.1. Cell Culture and Stimulation 

A549 cells (CCL-185) and PC-9 cells (a kind gift from Professor Sos (Institute of Pathology and Department of Translational Genomics, University Hospital Cologne)) were cultivated in RPMI-1640 (Sigma Aldrich, Steinheim, Germany) supplemented with 10% FCS (Bio&SELL GmbH, Nürnberg, Germany) and 2 mM L-glutamine, 100 U/mL potassium penicillin and 100 µg/mL streptomycin sulfate (all from Sigma-Aldrich). Cells were kept for four days in zinc-adequate (ZA), zinc-deficient (ZD) or zinc-deficient medium with 0.1 µM imatinib (Sigma Aldrich) (ZD+I) and zinc-supplemented (ZS) RPMI medium. The regular medium contained 7 µM zinc and was defined as zinc-adequate (ZA). For zinc supplementation, 50 µM zinc sulfate (Sigma Aldrich) was added to the ZA medium. Zinc-supplemented medium, thus, contained 57 µM zinc. Zinc-deficient medium was generated using Chelex 100 beads (Bio-Rad Laboratories Inc., Hercules, CA, USA) following the manufacturer’s instructions and contained 0.3 µM zinc. Other chelated cations were reconstituted as described before [65]. Medium zinc contents were measured using atomic absorption spectroscopy (AAS) as previously described [66]. 

Cells were stimulated with 10 ng/mL epidermal growth factor (EGF, Immunotools, Friesoythe, Germany) if not indicated differently and 5 ng/mL transforming growth factor alpha (TGF-α, Pepro-Tech, Cranbury, NJ, USA) if not indicated differently for 30 min, one hour or six hours. 

### 4.2. Western Blotting

After stimulation, cell pellets were resuspended in sample buffer as previously described [33], sonicated for 10 s and heated at 95 °C for 3–5 min to ensure protein denaturation. Samples were separated using sodium-dodecyl-sulfate polyacrylamide gel electrophoresis (8.5% polyacrylamide gel) next to a colored protein standard on each gel (New England Biolabs, Frankfurt, Germany). Samples were then blotted to a nitrocellulose membrane (Amersham, Piscataway, NJ, USA), then blocked for at least one hour in tris-buffered saline (TBS-T: 20 mM Tris-HCl, 136 mM NaCl (Applichem, Darmstadt, Germany)), supplemented with 0.1% Tween 20 (Sigma-Aldrich) with 5% non-fat dry milk (Saliter, Obergünzburg, Germany) and afterwards incubated in primary antibody overnight at 4 °C. Antibodies were bought from Cell Signaling Technologies (Danvers, MA, USA) and used as recommended by the manufacturer: EGFR: #4267; phosphorylated Y1068 EGFR: #3777; β-actin: #4967; E-cadherin: #3195; PD-L1: #13684. Incubation with secondary anti-rabbit antibody (Cell Signaling Technologies) occurred for two hours at room temperature and the antibody was diluted in TBS-T supplemented with 5% non-fat dry milk. Subsequently, luminol enhancer solution and peroxide (Westar Antares, Cyanagen, Bologna, Italy) were added to the membranes for five minutes. Chemiluminescence was detected with LAS-3000 (Fujifilm Lifesciences, Düsseldorf, Germany) and quantified using ImageJ (National Institutes of Health, Bethesda, MD, USA).

### 4.3. Flow Cytometry 

Stimulated or unstimulated A549 and PC-9 cells were stained with anti-human Alexa Fluor 647-coupled EGFR antibody or Alexa Fluor 647 mouse IgG1 (both Biolegend, San Diego, CA, USA) as previously described [33]. PBMC from co-cultures were stained with anti-human FITC-coupled CD3 antibody or FITC-coupled mouse IgG1 (both BD Biosciences) for 20 min. Subsequently, propidium iodide (10 μg/mL) was added for an additional 10 min. All samples were measured with flow cytometry (FACScalibur, BD Sciences, Heidelberg, Germany) and data were analyzed with Cellquest Software 3.0 (BD Sciences). Illustrations of the gating strategy can be found in Supplemental Figure S1. 

### 4.4. Measurement of Intracellular Labile Zinc Concentration 

Cells were loaded with 5µM ZinPyr-1 (4′,5′-bis[[bis(2-pyridinylmethyl) amino] methyl]-2′,7′-dichloro-3′,6′-dihydroxy-spiro[isobenzofuran-1(3H),9′-[9H]xanthen]-3-one, Santa Cruz, Dallas, TX, USA), a cell-permeating, zinc-selective fluorescent probe in phosphate-buffered saline (PBS from Sigma-Aldrich) and incubated for 30 min at 37 °C, then washed twice with PBS. Total fluorescence (F) levels of stained cells were measured without any further treatment. To obtain values for minimal (Fmin) and maximal (Fmax) fluorescence to calculate the total free intracellular zinc concentration, 50 µM of the zinc chelator TPEN (N,N,N′,N′-tetrakis(2-pyridinylmethyl)-1,2-ethanediamine) for Fmin or 100 µM zinc and 5 µM of its ionophore pyrithione (all from Sigma Aldrich) were added to the cells and incubated for 15 min at 37 °C, then measured immediately afterwards with flow cytometry (FACScalibur, BD) as previously described [67]. The intracellular zinc concentration was calculated with the dissociation constant of the ZinPyr-1–zinc complex (0.7 nM) and the following formula:

$$\mathrm{intracellular}\;\mathrm{labile}\;\mathrm{zinc}\;[nM]=kd*\frac{\left(F-Fmin\right)}{\left(Fmax-F\right)}.$$

### 4.5. RNA Isolation and cDNA Reverse Transcription 

RNA isolation from A549 cells proceeded with the EXTRACTME total RNA KIT (Blirt, Gdansk, Poland) according to the manufacturer’s instructions. The total RNA content was then measured using a NanoDrop ND 1000 spectrophotometer (Thermo Fisher Scientific, Maltham, MA, USA). Here, 1 µg RNA was reverse-transcribed with the qScript cDNA synthesis kit (Quantabio, Beverly, MA, USA) following the manufacturer’s protocol. The resulting cDNA was used for SYBR-green-based qRT-PCR (Quant Studio 3, Thermo Fisher). Primer sequences and temperatures were used as follows: GAPDH forward primer 5′GAA GGT GAA GGT CGG AGT C3′ and reverse primer 5′GAAGAT GGTGATGGGATTTC3′ at 60 °C for 60 s; E-cadherin forward primer 5′AGTGCCAACT GGACCATTCA3′ and reverse primer 5′TCTTTGACCACCGCTCTCCT3′ at 55 °C for 60 s then 72 °C for 60 s; PD-L1 forward primer 5′GGTGCCGACTACAAGCGAAT3′ and reverse primer 5′GGTGACTGGATCCACAACCAA3′ at 60 °C for 30 s; MMP-2 forward primer 5′AGATCTTCTTCTTCAAGGACCGGTT3′ and reverse primer 5′GGCTGG TCAGTGGCTTGGGGTA3′ at 64 °C for 60 s then 72 °C for 30 s; ZIP 8 forward primer 5′CCT CGG ATT GAT TTT GAC TCC ACT3′ and reverse primer 5′AGC AGG ATT TGC ATA GCA TGT CAC3′ at 60 °C for 60 s; ZIP10 forward primer 5′TAG CCG TCT GTC ATG AAC TGC3′ and reverse primer 5′TCA TAG AGG GCA ATC ACC AGC ATA3′ at 60 °C for 60 s; MT1,2 primers were used as described before [39] with forward primer 1 5′GCA CTT CGT GCA AGA AAA GCT-3′, forward primer 2 5′GCA CCT GCA AGA AGA GCT3′ and reverse primer 1 5′GCA GCC TTG GGC ACA CTT3′, reverse primer 2 5′GCA GCC CTG GGC ACA CTT3’ at 60 °C for 45 s; ZnT1 forward primer 5′GGC CAA TAC CAG CAA CTC CAA3′ and reverse primer 5′TGC AGA AAA ACT CCA CGC ATG T3′ at 60 °C for 60 s. PCR results were then analyzed using the ΔΔCT method, whereby GAPDH served as the housekeeping gene.

### 4.6. Co-Cultures 

A549 cells were kept in ZA, ZD, ZD+I or ZS media as described above for three days. After three days, media were discarded. After written consent and ethics committee approval (RWTH Aachen University Hospital, document No.: EK 023/05), healthy volunteers donated blood. PBMC (peripheral blood mononuclear cells) were isolated from whole blood of healthy volunteers using Ficoll density centrifugation as described before [68], and cell numbers were adjusted to 1 × 10^6^/mL in zinc-adequate medium. PBMC were added to the A549 cells and co-cultured following Chen et al. [30] for another three days, then harvested for further examination.

### 4.7. Assay of Membrane Fluidity 

Membrane fluidity was measured with the membrane fluidity kit (ab189819) from Abcam (Cambridge, UK) as described before [44], by detecting changes in the spectral properties of 1-pyrendecanoidacid using a SPARK10 multi-mode reader (Tecan, Männedorf, Switzerland).

### 4.8. MTT Assay 

The metabolic activity levels of zinc-adequate, -deficient and -supplemented A549 cells were measured as described before [67]. The reduction of the tetrazolium dye MTT (3-(4,5-dimethylthiazol-2-yl)-2,5-diphenyltetrazolium bromide) to its insoluble formazan by cellular NAD(P)H oxidases was measured using a SPARK10 multi-mode reader (Tecan).

### 4.9. Statistical Analysis 

Statistical analysis was performed using GraphPad-Prism (software version 9, GraphPad Software, San Diego, CA, USA). As a statistical test for more than two data sets, one-way ANOVA with Tukey’s post hoc test was applied; if values were missing for random reasons, data sets were analyzed by fitting a mixed model. Multiple *t*-tests were applied for single comparisons. Statistically different data sets were assumed at a *p*-value below 0.05. Statistical significances are depicted as * (*p* < 0.05) and ** (*p* < 0.01) for multiple *t*-tests and with different letters for one-way ANOVA. The same letters are never shared by significantly different data sets.

## Data Availability

The data presented in this study are available on request from the corresponding author.

## Supplementary Materials

The following supporting information can be downloaded at: https://www.mdpi.com/article/10.3390/ijms23042270/s1.
