# Supplementary material for: Zinc Status Impacts the Epidermal Growth Factor Receptor and Downstream Protein Expression in A549 Cells
Source: Int J Mol Sci. 2022 Feb 18;23(4):2270. doi: 10.3390/ijms23042270 (PMC8876057; doi:10.3390/ijms23042270)

Supplemental Material

Supplemental Figure S1 Gating strategies for FACS analyses

A Measurement of the EGFR surface expression of A549 cells

Vital A549 cells were determined by their typical appearance in the forward and site scatter and gated. The histograms show the measurements of the mean fluorescence intensity of the isotype control and the EGFR antibody for the gated cells.

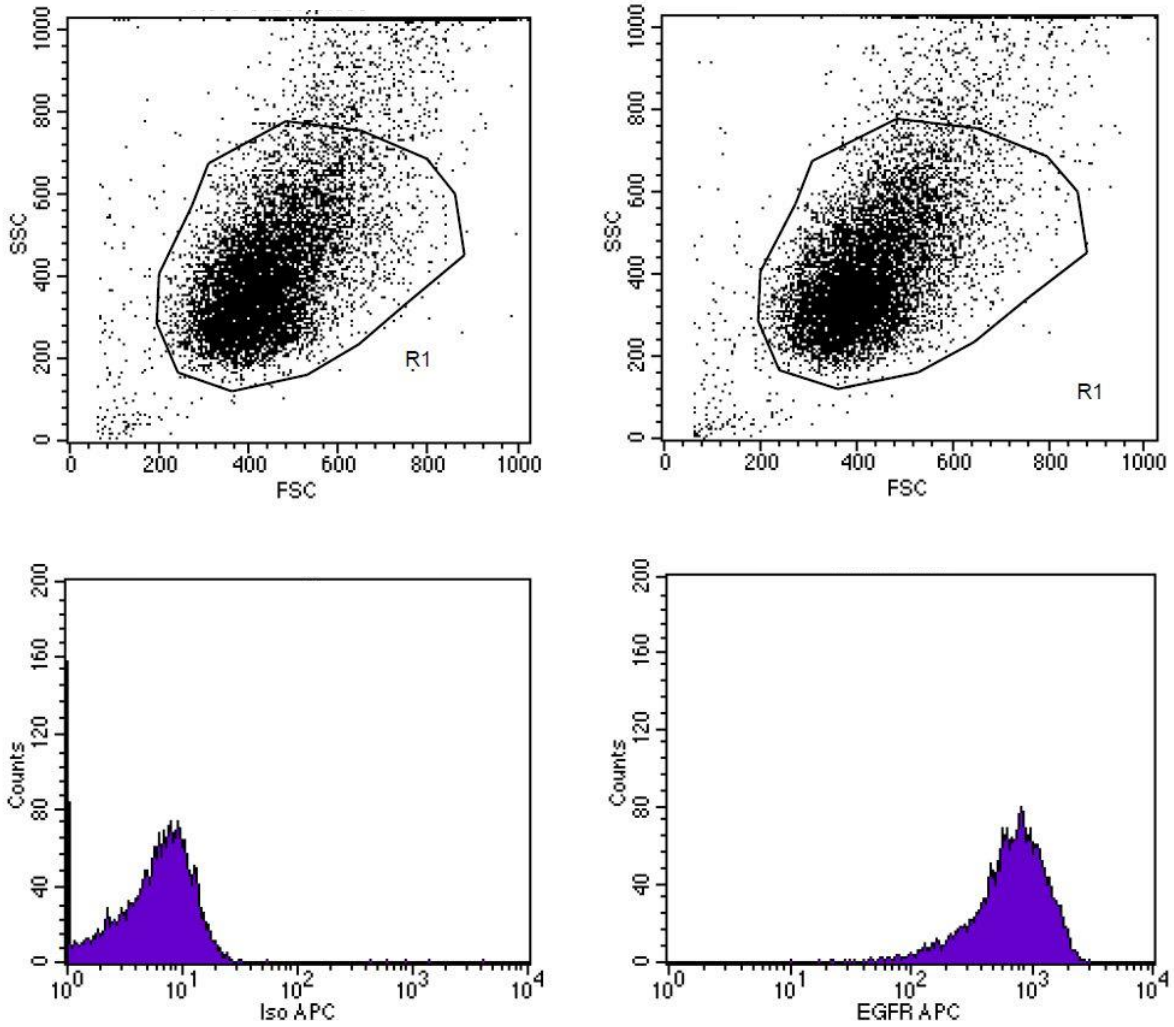

B Vitality analysis of CD3+ T cells

To Assess vitality of CD3+ T-cells, debris was excluded (R1) and T cells were gated according to high CD3 expression (R2). Mean fluorescence intensity for propidium iodide (PI) of CD3+ cells was recorded and MFI above a certain threshold was counted as PI positive cells (M1)

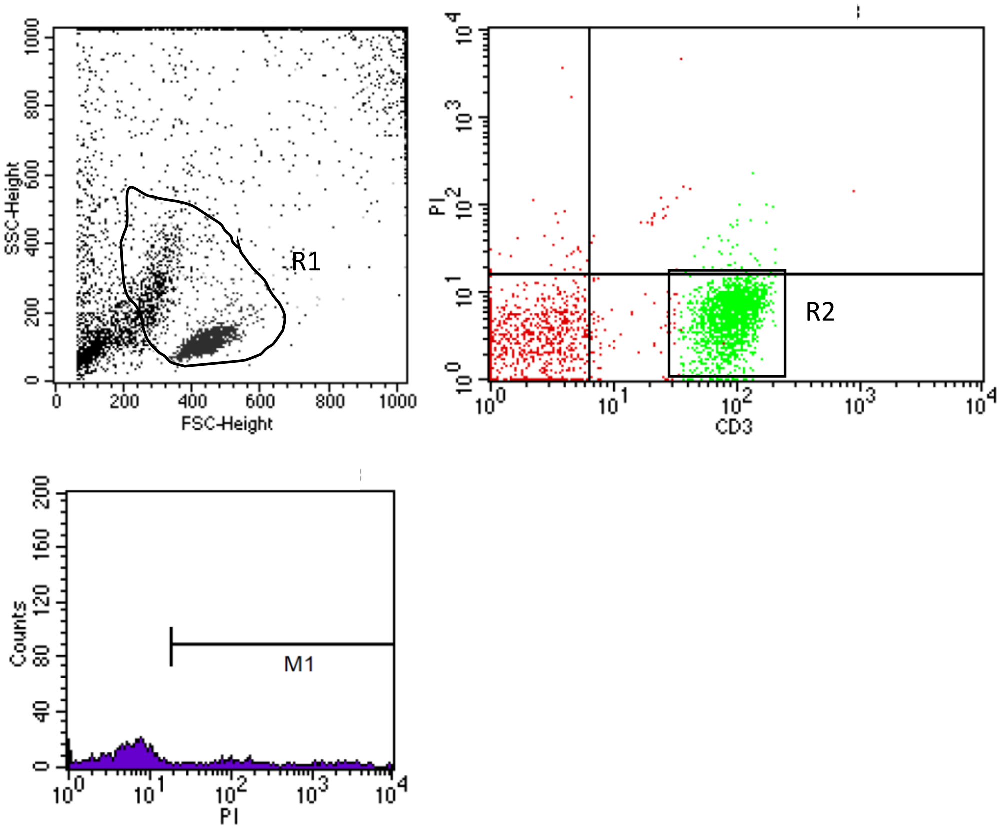

Supplement: Supplementary file 1 [file ijms-23-02270-s001.zip › ijms-1510534-supplementary.pdf]
